# Psychiatric risk and resilience: Plasticity genes and positive mental health

**DOI:** 10.1002/brb3.2137

**Published:** 2021-05-01

**Authors:** Paul G. Nestor, Victoria Choate Hasler, Keira O'Donovan, Hannah E. Lapp, Sara B. Boodai, Richard Hunter

**Affiliations:** ^1^ Department of Psychology University of Massachusetts Boston Boston MA USA; ^2^ Laboratory of Neuroscience Harvard Medical School Boston MA USA; ^3^ Laboratory of Neuroendocrinology The Rockefeller University New York NY USA

**Keywords:** at‐risk mental state, personality, plasticity genes, positive mental health, stress

## Abstract

**Objective:**

The at‐risk mental state (ARMS) for psychosis has long played a key role in diathesis‐stress models of schizophrenia. More recent studies, however, have called for extending the boundaries of the ARMS construct beyond attenuated psychosis in nonhelp‐seeking samples to include not only other vulnerability indicators but also protective factors related to genotype, mental health, personality, and cognition.

**Method:**

Accordingly, we assessed in a sample of 100 college students, the ARMS construct with the Brief Prodromal Questionnaire (PQ‐B) for psychosis, in conjunction with measures of positive mental health, childhood adversity, psychiatric symptoms, personality traits, social cognition, and genetic variables derived from assays of the serotonin transporter (5‐HTTLPR) and the brain‐derived neurotrophic factor (BDNF).

**Results:**

Higher PQ‐B scores correlated positively with vulnerability indicators of childhood adversity and heightened levels of a wide variety of psychiatric symptoms but correlated negatively with protective factors of better overall mental health, social cognition as well as with a distinct NEO profile marked by reduced neuroticism and elevated agreeableness and conscientiousness. Multivariate analyses indicated that a composite ARMS measure comprised of PQ‐B scores plus anxiety and depression symptoms revealed significant genotype differences, with lowest risk and highest resilience for allelic carriers of 5‐HTTLPR‐short and BDNF Met polymorphisms.

**Conclusions:**

Results provided support for extending the ARMS construct, pointing to important contributions of personality, social cognition, and genes that support neural plasticity in mitigating vulnerability and enhancing resilience and well‐being.

## Graphical abstract

We examined the psychotic risk in relation to positive mental health and plasticity genes in a healthy college student sample. Lower psychotic risk correlated with higher positive mental health and a particular set of plasticity genes. Results point to important contributions of positive mental health and genes that support neural plasticity in mitigating general psychiatric risk and enhancing well‐being.

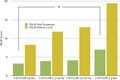

## 1 INTRODUCTION

The concept of risk or vulnerability has long played a critical role in both mental health research and practice. Its historical roots may be traced to the early work of Harry Stack Sullivan (1927) who first described the prodromal state of schizophrenia as characterized by subclinical symptoms and signs of psychosis (Kirch et al., 1992). The subsequent identification of the so‐called schizophrenia prodrome held the promise of providing a window for targeted treatment and prevention, an invaluable opportunity for therapeutic intervention during a presumed critical period that too often had been viewed solely as an initial step in an inevitable disease progression. No doubt inspired by the potential clinical impact of these early efforts, more contemporary studies of the past 15 years have developed rigorous measures of the schizophrenia prodrome, now cast as an at‐risk mental state (ARMS) that can be reliably and validly assessed with structured interview, self‐report, or combination thereof (Fusar‐Poli et al., 2012) In fact, meta‐analyses of longitudinal studies showed, independent of risk instrument, individuals assessed as at‐risk had transition rates to psychosis of 18% after 6‐month follow‐up, 22% after 1 year, 29% after 2 years, and 36% after 3 years (Fusar‐Poli et al., 2012).

Recent studies have called for extending the boundaries of the ARMS construct beyond attenuated psychosis to include other psychiatric symptoms such as depression and anxiety as well as other measures of stress and adversity in nonhelp‐seeking samples (Lee et al., 2018; Linscott & Van Os, 2013; van Os, & Murray, 2013; van Os & Reininghaus, 2016). From this transdiagnostic perspective, ARMS may be viewed as a dynamic and malleable condition, which might remit, persist without worsening, or diverge along several different illness trajectories (McGorry et al., 2018; McGorry & Nelson, 2016). While the factors that may influence etiology and ultimate outcome or resolution remain an active area of research, risk models have traditionally emphasized a diathesis‐stress framework in which vulnerability for developing a disorder can emanate from biological, psychological, or sociocultural sources triggered by adverse environmental events or experiences perceived as overwhelming one's personal resources (e.g., Hooley et al., 2017; Nolen‐Hoeksema & Watkins, 2011). For example, studies have shown that individuals with a particular genetic diathesis when exposed to environmental adversity are more likely to develop major depression than those who experience the same level of major life stressors but without the genetic vulnerability (Caspi et al., 2003).

More recent formulations, however, have provided a new perspective on risk, one less psychopathological in focus and more resonant with positive psychology, examining what may be viewed as the “bright side” of these gene–environment interactions (Ellis & Boyce, 2011). Known as the differential‐susceptibility hypothesis, it proposes that the very same characteristics such as a “risky” genotype or a “reactive” temperament that make individuals disproportionately vulnerable to stress also make them disproportionately more likely to benefit from positive experiences and environmental supports (Belsky & Hartman, 2014). Here, vulnerability genes are reconceptualized as plasticity alleles that confer sensitivity to both positive and negative experiences and environments. Unknown, however, is whether such variation in plasticity or malleability “for better and for worse” contributes to individual differences in mental health in mitigating or protecting against clinical risk.

Accordingly, adopting a positive psychology framework (see e.g., Layous et al., 2014), we examine the concept of ARMS through the lens of plasticity, broadly defined, in relation to genotype, mental health, personality, and neuropsychological functioning. We targeted specific allelic variants arising from a priori selected, single nucleotide polymorphisms (SNPs) in two candidate plasticity genes, serotonin (5‐HTTLPR) and neurotrophic factor (BDNF), each linked to individual differences in emotionality, stress, cognition, and personality (Belsky & Hartman, 2014). In so doing, single and polygenic effects of these alleles on ARMS can be directly tested in conjunction with measures of childhood stress and current levels of psychiatric vulnerability, positive mental health, personality traits, and neuropsychological functioning. Together, these psychological and biological measures provide a multimethod design to test the unifying hypothesis that reduced ARMS may be expressed genetically, by increased plasticity alleles, and, behaviorally, by higher mental health, specific personality profiles and better neuropsychological performance.

## 2 MATERIALS AND METHODS

### 2.1 Participants

One hundred participants, recruited from the greater Boston area, primarily at the University of Massachusetts, Boston (UMB) were between the ages of 18 and 25 (*M* = 21.22 years, *SD* = 1.99) and identified as English speaking for at least 5 years prior to study enrollment. Seventy percent of participants identified as biologically female, 42% racially identified as White, 72% reported the United States of America as their country of origin, and 63% endorsed 1–3 years of college as their level of education. The Institutional Review Board at University of Massachusetts Boston approved all research study procedures. Consenting participants completed self‐report measures and neuropsychological tests, and then provided a DNA sample via a cheek swab for the assaying of genotypes. Participants were compensated $25 for their time or received extra credit in psychology courses.

### 2.2 Self‐report measures

*Prodromal Questionnaire‐Brief* (PQ‐B). The PQ‐B is a 21‐item self‐report questionnaire designed to assess the presence or absence of psychosis‐risk syndromes (Loewy et al., 2011). Based on the items from the Structured Interview for Prodromal Syndromes (McGlashan et al., 2001), the PQ‐B assesses positive symptoms of psychosis experienced in the past month PQ‐B has been shown to be an effective and efficient instrument for screening psychosis risk across a wide variety of samples and settings, including Chinese‐speaking mental health referrals (Xu et al., 2016), Nigerian secondary school students (Okewole et al., 2015), Dutch‐speaking young adults ages 18–35 recruited from a general help‐seeking population (Ising et al., 2012), and male prisoners (Jarrett et al., 2015).

*Brief Symptom Inventory* (BSI). The BSI is a 53‐item scale that measures psychiatric symptoms status across nine distinct domains: Somatization, Obsessive–Compulsive, Interpersonal Sensitivity, Anxiety, Hostility, Depression, Paranoid Ideation, Psychoticism, and Phobic Anxiety (Derogatis & Spencer, 1982). The BSI also includes measures of overall Global Severity Index (GSI) and a Positive Symptom Distress Index (PSDI). BSI scores reflect current psychiatric status on a Likert scale ranging from 0 (not at all) to 4 (extremely). The BSI has demonstrated good internal consistency among nonpsychiatric populations (0.71–0.85 across scales) and moderate to high test–retest reliability (0.68–0.91 across scales) and convergent and discriminant validity with the Minnesota Multiphasic Personality Inventory (MMPI)(Derogatis & Melisaratos, 1983; Derogatis & Spencer, 1982).

*Adverse childhood experiences* (ACE). This scale is a 10‐item measure that assesses eight categories of adverse experiences in childhood, including emotional, physical, and sexual abuse, and household dysfunction (i.e., substance abuse, mental illness, mother treated violently, and incarcerated household member). Participants are asked to provide “Yes” or “No” responses to each of the 10 items. Scores range from 0 to 10, with higher scores indicative of greater number of adverse events in childhood (Anda et al., 2006).

*Mental health continuum‐short form* (MHC‐SF). Developed by Keyes (2005) the 14‐item MHC‐SF addresses three components of well‐being (emotional, psychological, and social). Respondents rate each item on a Likert scale ranging from 1 (low frequency) to 5 (high frequency), with scores ranging from 14 to 70, higher indicative of better mental health. The MHC‐SF also provides predefined cutoff scores to distinguish three levels of mental health: (flourishing, moderately mentally healthy, and languishing). Studies report strong psychometric properties for the MHC with estimates of internal reliability of 0.89 (Lupano Perugini et al., 2017) and 0.91 across adolescent and adult sample with various cultural contexts including Canada, the United States, Netherlands, Argentina, Korea, Poland, India, and Italy (see Luijten et al., 2019 **)**.

*Revised NEO personality test* (NEO‐PI‐R). The NEO‐PI‐R is an objective, self‐report measure of five distinct and presumably universal personality traits: neuroticism, extraversion, openness, agreeableness, and conscientiousness (Costa & McCrae, 1992). DeYoung et al. (2010) reported alpha reliabilities of internal consistency for the five NEO trait scales as 0.92 for Neuroticism; 0.87 for Extraversion; 0.89 for Openness; 0.91 for Agreeableness; and 0.91 for Conscientiousness.

#### 2.2.1 Neuropsychological measures

Reading the mind's eyes test (RMET; Baron‐Cohen et al., 1997), used as a computerized task of social cognition, specifically Theory of Mind, consists of 36 trials, presented in randomized order, with each trial displaying a black‐and‐white photograph of the eyes, eyebrows, and bridge of the nose of a White male or female individual making a facial expression, and below the photograph are four adjectives that describe a complex emotion (e.g., reflective, aghast, irritated, and impatient). Test–retest reliability has been reported in nonclinical populations to range from 0.7 to 0.8 (Hallerbäck et al., 2009), and the RMET has been shown to correlate positively with measures of empathy (see: Lawrence et al., 2004), and to distinguish social interaction disturbances in clinical populations, such as Autism Spectrum Disorders, (Baron‐Cohen et al., 1997). Standardized cognitive neuropsychological tests were oral reading subtest of the Wide Range Achievement Test third edition (WRAT3; Wilkinson, 1993), Trail Making Test (Arbuthnott & Frank, 2000), both Trails A and Trails B (Lezak et al., 2012), Wechsler Adult Intelligence Scale‐Fourth Edition (WAIS‐IV; Wechsler, 2008) Coding subtest, and Wechsler Adult Intelligence Scale‐third edition (WAIS‐III; Wechsler, 1997) Digit Span subtest.

### 2.3 DNA collection and extraction

Cytobrush swabs (Coopersurgical Inc.) were used to collect buccal cells. Participants were instructed to brush the swab 30 times against the inside of their cheek while slowly rotating the swab. Swabs were immediately placed on ice and stored at −80 degrees C until DNA extraction. Buccal samples were extracted using a Zymo Quick DNA Universal Kit per the manufacturer's instructions (Zymo Research). DNA yield from buccal samples ranged from 0.48μg to 14.4μg of DNA. Extracted DNA was stored in molecular biology‐grade water at −80°C until genotyping analysis.

#### 2.3.1 5‐HTTLPR genotyping

Genotyping for 5‐HTTLPR polymorphisms was performed using polymerase chain reaction and resolution using gel electrophoresis (adapted from Smith et al., 2004). 25 μl PCR reactions were set up to contain 1X Green GoTaq Flexi Buffer, 1.5 mM MgCl_2_, 0.25 mM PCR Nucleotide Mix, 2.5 ng of DNA sample, and 0.15 μM of both forward and reverse primers (FW: 5′TGA ATG CCA GCA CCT AAC CC 3′ and RV: 5′TTC TGG TGC CAC CTA GAC GC 3′). DNA amplification was achieved used the following thermocycler programming: Initial denaturation was run for 11 min at 95°C, followed by 40 cycles of 45 s at 95°C, 45 s at 60°C, 45 s at 72°C, and a final elongation step of 72°C for 10 min. The two amplicon products varied by 44 base pairs (515 base pairs for the long allele and 471 base pairs for the short allele) and were visualized by running the DNA samples on a 1.5% agarose gel stained with 1.5% Ethidium Bromide. Length of amplicon was determined by comparing sample bands to a reference DNA ladder (ref: G695A; Promega) using Molecular Imaging ChemiDoc XRS+. Heterozygous 5‐HTTLPR genotype was visibly detected by the presence of two bands in the lane approximately 44 base pairs apart.

#### 2.3.2 BDNF genotyping

TaqMan SNP genotyping was used to determine BDNF val66met genotype (rs6265). 25 μl PCR reactions were performed using a predesigned 1X Taqman allelic discrimination assay (assay number: C__11592758_10; Applied Biosystems), containing forward and reverse primers and allele‐specific probe with 5ng of sample DNA. Genotypic amplification was achieved using the StepOne Plus Real‐Time (Applied Biosystems) PCR System with programming as follows: 95°C for 10m, followed by 42 cycles of 95°C for 15 s and 60°C for 1m. Genotype was determined from the resulting allelic discrimination plot.

For the BDNF gene, there were 72 Val/Val, 21 Met/Met, and 7 Val/Met carriers. We grouped Met/Mets (*n* = 21) with Val/Mets (*n* = 7) to form a “Met” carriers group (*n* = 28), with the remaining participants categorized as “Val/Val” (*n* = 72) genotype. For the 5‐HTTLPR transporter gene, there were 41 Long/Long, 38 Short/Long, and 21 Short/Short carriers. The distribution of genotypes followed the Hardy–Weinberg equilibrium for 5‐HTTLPR and BDNF alleles. In addition, we assigned the 41 Long/Long alleles to a 5‐HTTLPR transporter‐long (“5‐HTTLPR‐L” *n* = 41) group, and the remaining 38 Short/Long and 21 Short/Short carriers to a 5‐HTTLPR transporter short (“5 HTTLPR‐S” *n* = 59) group. Following previous research (Grabe et al., 2012), we further divided the 100 participants into four allelic groups: 1) 34 5‐HTTLPR‐L, BDNF Val/Val carriers; 2) 7 HTTLPR–L, BDNF Met carriers; 3) 38 5‐HTTLPR‐S, BDNF Val/Val carriers; and 4) 21 5HTTLPR‐S, BDNF Met carriers (see also, Nestor et al., 2019).

### 2.4 Statistical analysis

For the behavioral measures, Pearson correlations tested for associations of PQ‐B scores with ACE, NEO, MHC‐SF, RMET, and cognitive neuropsychological tests. We then performed to a mixed‐model 2 × 5 ANOVA with PQ‐B group as the between‐subjects factor, median split in two levels (low, high), and NEO personality traits as the within‐subjects factor with five levels (neuroticism, extraversion, openness, agreeableness, and conscientiousness). For the genetic data, *t* tests compared PQ‐B scores for BDNF (Val/Val, Met) and 5‐HTTLPR (long, short) groups. For multi‐group comparisons, we submitted PQ‐B scores to a 2 × 2 ANOVA with two between‐subjects factors of BDNF (Val/Val, Met) and 5‐HTTLPR serotonin transporter gene (long, short). This ANOVA tested for the main effects of BDNF and 5‐HTTLPR as well as for the interaction of BDNF × 5‐HTTLPR on PQ‐B scores. Finally, as a further test of the hypothesis for an extended ARMS phenotype, we submitted PQ‐B scores along with BSI anxiety and depression measures to a 2 × 2 multivariate analysis of variance (MANOVA) with two between‐subjects factors of serotonin (long, short) and BDNF (Val/Val, Met).

## 3 RESULTS

Participants (*N* = 100) reported on the PQ‐B an average of 4.08 (*SD* = 4.11) positive symptoms of psychosis in the past month with a mean level of distress of 12.06 (*SD* = 14.21). For the BSI, the sample had highest average *T*‐scores for Psychoticism (*M* = 61.72, *SD* = 11.96), Obsessive–Compulsive (*M* = 61.49, *SD* = 12.66), and Depression (*M* = 60.45, *SD* = 11.12) and for the NEO, neuroticism (*M* = 53.19, *SD* = 12.17), extraversion (*M* = 50.33, *SD* = 12.20), openness (*M* = 57.05, *SD* = 10.75), agreeableness (*M* = 49.93, *SD* = 11.41), and conscientiousness (*M* = 48.88, *SD* = 12.26). For the MHC‐SF, scores ranged from 13 to 70 (*M* = 46.24, *SD* = 13.49). For the ACE, participants reported on average 2.17 (*SD* = 2.31) exposures to adverse childhood experiences (see Table 1).

**TABLE 1:** Descriptive data for entire sample and single genotype groups

Measures	Entire sample (*n* = 100)	Serotonin long (*n* = 41)	Serotonin short (*n* = 59)	BDNF val (*n* = 72)	BDNF met (*n* = 28)
Brief Prodromal Questionnaire					
Symptoms	4.08 ± 4.12	4.64 ± 4.05	3.81 ± 4.16	4.04 ± 4.16	4.18 ± 4.06
Distress	12.06 ± 14.21	13.12 ± 12.95	11.32 ± 15.30	12.44 ± 14.99	11.07 ± 12.18
Brief Symptom Inventory^a^					
Somatization	55.32 ± 11.83	54.90 ± 10.43	55.61 ± 12.79	56.04 ± 11.88	53.46 ± 11.70
Obsessive–Compulsive	61.49 ± 12.66	61.71 ± 12.37	61.34 ± 12.95	61.82 ± 12.49	60.64 ± 13.28
Interpersonal sensitivity	59.79 ± 12.21	61.27 ± 10.94	58.76 ± 13.01	61.49 ± 12.33	55.43 ± 10.93
Depression	60.45 ± 11.12	61.34 ± 9.51	59.83 ± 12.15	61.89 ± 10.95	56.75 ± 10.87
Anxiety	56.42 ± 13.16	75.61 ± 11.71	55.61 ± 14.11	58.18 ± 13.24	51.93 ± 12.04
Hostility	57.07 ± 10.96	58.22 ± 10.28	56.27 ± 11.43	58.36 ± 10.83	53.75 ± 10.78
Phobic anxiety	57.40 ± 10.86	59.00 ± 10.62	56.29 ± 10.98	57.82 ± 11.40	56.32 ± 9.46
Paranoid ideation	58.33 ± 11.79	59.83 ± 11.10	57.29 ± 12.24	59.63 ± 11.80	55.00 ± 11.29
Psychoticism	61.72 ± 11.96	62.49 ± 11.64	61.19 ± 12.37	62.51 ± 12.36	59.68 ± 10.81
The NEO Personality Inventory^a^					
Neuroticism	53.19 ± 12.17	53.08 ± 9.80	53.26 ± 13.67	55.08 ± 12.22	48.00 ± 10.60
Extraversion	50.33 ± 12.20	48.97 ± 10.97	51.28 ± 13.01	48.25 ± 12.32	56.00 ± 10.06
Openness to Experience	57.05 ± 10.25	58.50 ± 9.76	56.04 ± 10.55	57.10 ± 10.08	56.92 ± 10.90
Agreeableness	49.93 ± 11.41	49.53 ± 10.80	50.21 ± 11.91	48.99 ± 11.35	52.50 ± 11.41
Conscientiousness	48.88 ± 12.26	49.68 ± 13.83	48.32 ± 11.11	47.73 ± 12.84	52.00 ± 10.08
Adverse childhood experiences (ACE)					
Total ACE	2.17 ± 2.31	2.63 ± 2.40	1.85 ± 2.20	2.26 ± 2.29	1.93 ± 2.37
Reading the mind in the eyes test (RMET)					
Total RMET	23.75 ± 4.45	23.43 ± 4.62	23.98 ± 4.35	24.03 ± 4.55	23.00 ± 4.14
Mental health continuum‐short form (MHC‐SF)					
Total MHC‐SF	46.21 ± 13.49	45.71 ± 15.01	46.61 ± 12.45	45.24 ± 14.11	48.82 ± 11.58
Neuropsychological measures					
WRAT‐3^b^	105.90 ± 15.49	101.73 ± 14.07	108.88 ± 15.89	106.79 ± 15.59	103.36 ± 15.24
Coding score^c^	11.11 ± 3.03	10.53 ± 2.77	11.53 ± 3.16	10.82 ± 2.94	11.92 ± 3.19
Longest digit forward^c^	9.51 ± 2.89	9.35 ± 3.25	9.61 ± 2.63	9.58 ± 3.01	9.31 ± 2.57
Longest digit backward^c^	9.82 ± 3.11	9.93 ± 3.57	9.75 ± 2.78	9.85 ± 3.31	9.77 ± 2.55
Trails A^d^	26.54 ± 12.08	26.75 ± 10.51	26.39 ± 13.17	27.12 ± 10.34	24.95 ± 16.04
Trails B^d^	67.32 ± 34.15	67.92 ± 34.48	66.91 ± 34.21	66.91 ± 32.35	68.45 ± 39.31

Abbreviation: BSI, Brief Symptom Inventory.

^a^ Descriptive data for BSI and NEO are *t*‐scores (*M* = 50, *SD* = 10).

^b^ Descriptive data for WRAT‐3 are standard scores (*M* = 100, *SD* = 15).

^c^ Descriptive data for longest digit forward, longest digit backward, and coding are scaled scores (*M* = 10, *SD* = 3).

^d^ Descriptive data for Trails A and B are times in seconds.

Table 2 presents the correlations of PQ‐B scores with behavioral measures. As seen in Table 2, PQ‐B scores, indicative of greater psychotic risk, correlated significantly (all *p*'s < .001) with increased psychiatric symptomatology, across all nine BSI domains as well as lower mental health, as assessed by the MHC‐SF. Likewise, higher number of reported adverse childhood experiences correlated significantly with increased PQ‐B scores for total symptoms, *r* = .294, *p* = .003, and for distress level, *r* = .300, *p* = .002. In addition, for performance‐based measures, lower RMET scores correlated with increase PQ‐B risk for both total symptoms *r* = −.219, *p* = .032 and for level of distress, *r* = .203, *p* = .04. By contrast, PQ‐B scores did not correlate with any of the cognitive neuropsychological measures.

**TABLE 2:** Correlations of PQ‐B scores with BSI, NEO, and ACE measures

MEASURES—Domain total and index scores	PQ‐B symptoms (*n* = 100)	PQ‐B distress (*n* = 100)
Brief Symptom Inventory		
Somatization	0.595**	0.584**
Obsessive–Compulsive	0.557**	0.540**
Interpersonal sensitivity	0.523**	0.542**
Depression	0.487**	0.514**
Anxiety	0.578**	0.585**
Hostility	0.585**	0.574**
Phobic anxiety	0.592**	0.624**
Paranoid ideation	0.584**	0.558**
Psychoticism	0.594**	0.589**
The NEO Personality Inventory		
Neuroticism	0.441**	0.507**
Extraversion	−0.256*	−0.322**
Openness to experience	0.006	−0.022
Agreeableness	−0.258*	−0.264**
Conscientiousness	−0.311**	−0.344**
Adverse childhood experiences (ACE)		
Total ACE	0.294**	0.300**
Reading the mind in the eyes test (RMET)		
Total RMET	−0.219*	−0.203*
Mental health continuum‐short form (MHC‐SF)		
Total MHC‐SF	−0.514**	−0.529**
Neuropsychological measures		
WRAT	−0.121	−0.095
Coding	−0.121	−0.128
Longest digits forward	−0.078	−0.100
Longest digits backward	−0.148	−0.179
Trails A time in seconds	0.066	0.075
Trails B time in seconds	0.166	0.156

Abbreviations: BSI, BSI, Brief Symptom Inventory; PQ‐B, Brief Prodromal Questionnaire; WRAT, Wide Range Achievement Test.

* Correlation is significant at the .05 level (two‐tailed).

** Correlation is significant at the .01 level (two‐tailed).

For the NEO, as shown in Table 2, higher PQ‐B symptom scores correlated significantly with increased neuroticism, *r* = .441, *p* < .001, and lower levels of extraversion, *r* = −.256, *p* = .01, agreeableness, *r* = −.258, *p* = .01 and conscientiousness, *r* = −.311, *p* = .002. As a follow‐up to these correlational analyses, we performed ANOVA using the PQ‐B median (Median = 3, *SD* = 4.11) to divide the sample into high and low PQ‐B groups. Thus, a mixed‐model ANOVA with PQ‐B group as the between‐subjects factor with two levels (low, high) and NEO personality traits as the within‐subjects factor with five levels (neuroticism, extraversion, openness, agreeableness, and conscientiousness) revealed a highly significant PQ‐B group × personality interaction, *F*(4, 380) = 8.38, *p* < .001, partial eta squared = 0.081. This interaction reflected elevated neuroticism *t*(95) = −3.69, *p* < .001 and reduced levels of agreeableness *t*(95) = 2.87, *p* = .03 and conscientiousness, *t*(95) = 3.84, *p* < .001 for the high‐PQ‐B group relative to the low‐PQ‐B group.

As shown in Table 1, PQ‐B scores did not differ for 5‐HTTLPR serotonin transporter long (*n* = 41) versus short (*n* = 59) carriers, nor for BDNF Val/Val (*n* = 72) versus Met carriers (*n* = 28). Similarly, there were no single gene effects for either serotonin or BDNF on scores from ACE, BSI, RMET, and for the cognitive neuropsychological measures, with the sole exception of significantly higher scores in WRAT3 word reading for serotonin transporter short (*M* = 108.88, *SD* = 15.88) relative to long (*M* = 101.73, *SD* = 14.02) carriers, *t*(94) = −2.28, *p* = .025. For the NEO, significantly lower neuroticism for Met (*M* = 48.00, *SD* = 10.60) relative to Val/Val (*M* = 55.00, *SD* = 12.22) carriers, *t*(95) = 2.62, *p* = .01 as well as elevated extraversion for Met (*M* = 56.00, *SD* = 10.06) versus Val/Val (*M* = 48.25, *SD* = 12.32), *t*(95) = −2.87, *p* = .005.

**FIGURE 1:**
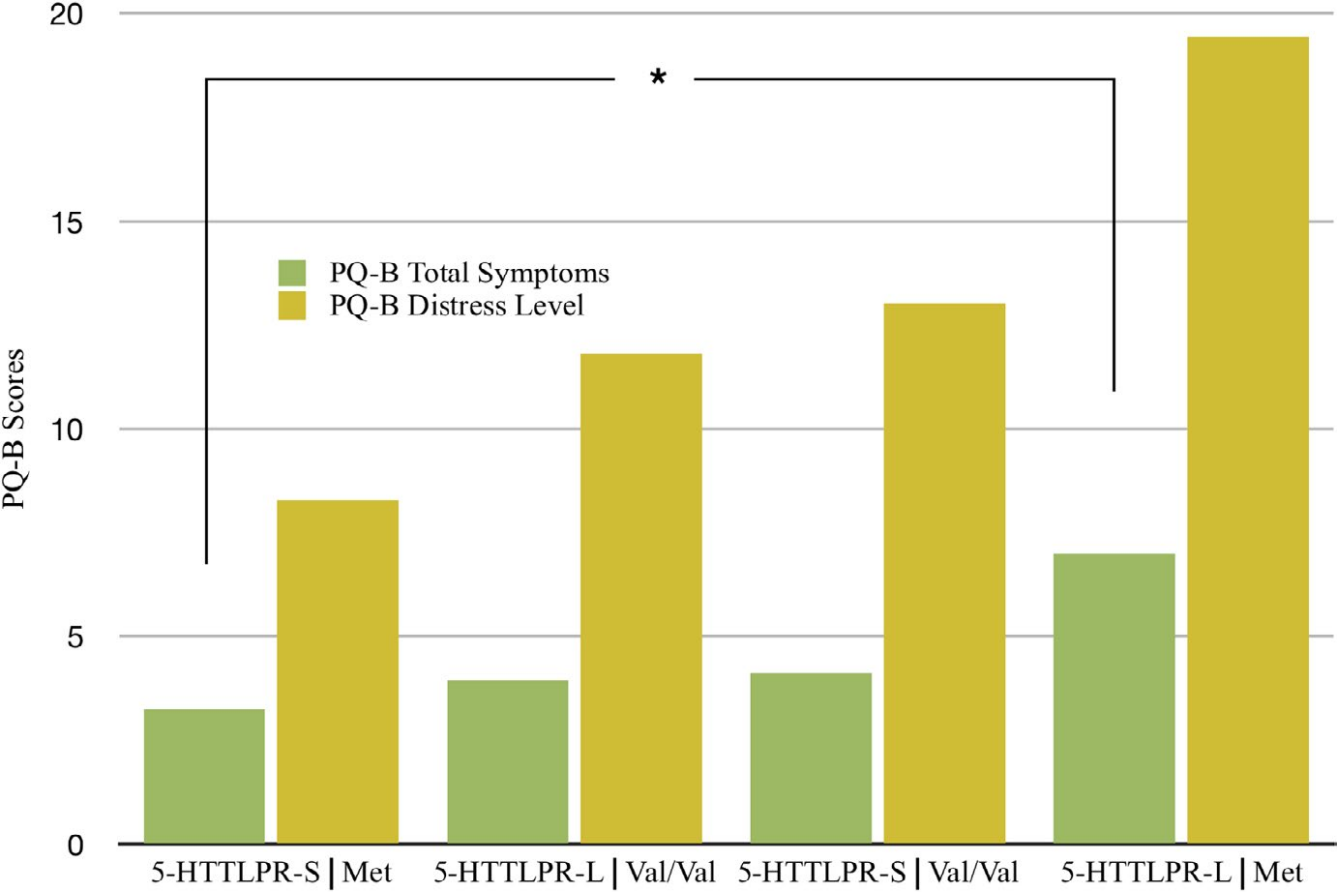
Brief Prodromal Questionnaire (PQ‐B) scores for the four allelic groups

To examine the effects of these two candidate genes simultaneously on ARMS, we submitted PQ‐B total symptoms scores to a 2 × 2 ANOVA with two between‐subjects factors of serotonin (long, short) and BDNF (Val/Val, Met). The 2 × 2 ANOVA revealed a statistically significant interaction of BDNF × serotonin, *F*(1, 96) = 3.82, *p* = .05, partial eta squared = 0.038. As shown in Figure 1, the effect of BDNF on PQ‐B symptom scores varied as a function of 5‐HTTLPR. 5‐HTTLPR‐S, Met (*n* = 21) carriers had the lowest PQ‐B symptom score (*M* = 3.24, *SD* = 3.21) scores, differing significantly from 5‐HTTLPR‐L, Met (*n* = 7) carriers who recorded the highest PQ‐B (*M* = 7.00, *SD* = 5.23) score, *t*(26) = 2.29, *p* = .03. 5‐HTTLPR‐S. Val/Val (*n* = 38) carriers had the next highest PQ‐B symptom score (*M* = 4.13, *SD* = 4.62) followed by 5‐HTTLPR‐L, Val/Val (*n* = 34) carriers (*M* = 3.94, *SD* = 3.64), but this group difference did not achieve statistical significance. Heightened PQ‐B scores for 5‐HTTLPR‐L, Met carriers (*n* = 7) in comparison with 5‐HTTLPR‐L, Val/Val carriers (*n* = 34) approached statistical significance, *t*(39) = −1.88, *p* = .068.

**FIGURE 2:**
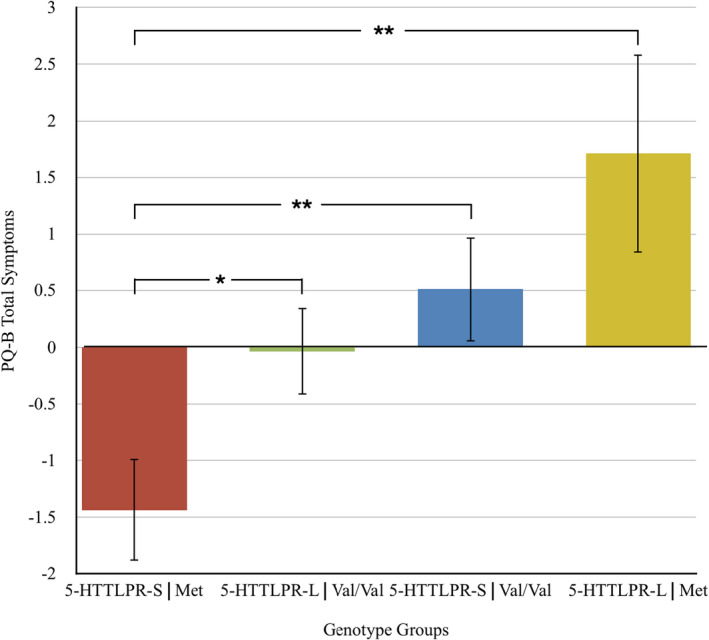
Composite at‐risk mental state *z*‐scores for the four allelic groups. PQ‐B, Brief Prodromal Questionnaire

Finally, as a further test of the hypothesis for an extended ARMS phenotype, we submitted PQ‐B total symptoms scores along with BSI anxiety and depression measures to a 2 × 2 MANOVA with two between‐subjects factors of serotonin (long, short) and BDNF (Val/Val, Met). MANOVA revealed a highly statistically significant multivariate interaction effect of BDNF × serotonin, *F*(3, 94) = 4.15, *p* = .008, partial eta squared = 0.117. Follow‐up univariate tests revealed a highly statistically significant interaction for depression, *F*(1, 96) = 12.39, *p* = .001, partial eta squared = 0.114, with more modest but still statistically significant contributions for anxiety, *F*(1, 96) = 5.30, *p* = .025, partial eta squared = 0.052 and PQ‐B, *F*(1, 96) = 3.82, *p* = .05, partial eta squared = 0.038. Figure 2 presents a composite ARMS *z*‐score comprised of PQ‐B symptoms plus BSI anxiety and depression measures. As shown in Figure 2, allelic carriers of 5‐HTTLPR‐short and BDNF Met polymorphisms had lowest composite ARMS Z‐score (*M* = −1.438, *SD* = 2.07), significantly different from 5‐HTTLPR‐L, Val/Val (*M* = −0.035, *SD* = 2.23), *t*(53) = 2.33, *p* = .023, 5‐HTTLPR‐S, Val/Val (*M* = 0.511, *SD* = 2.85), *t*(57) = 2.76, *p* = .008, and 5‐HTTLPR‐L, Met (*M* = 1.711, *SD* = 2.31), *t*(26) = 3.40, *p* = .002.

## 4 DISCUSSION

The current study investigated the ARMS construct broadly, focusing not only on traditional vulnerability indicators of symptoms and stressful events, but also on protective factors in the form of specific individual attributes, namely genotype, mental health, personality, social cognition, and neuropsychological performance. ARMS, as assessed by the PQ‐B correlated significantly with other psychiatric symptoms and childhood adversity, all key variables that have been previously linked to psychotic‐like experiences in prodromal studies of referrals to clinics specialized for early intervention (Fusar‐Poli et al., 2012). Of particular importance for external validity of the current study that relied on nonhelp‐seeking college student sample is that population‐based studies with children and adolescents have also reported similar associations between psychotic risk measures and symptoms and cognitive changes (Karcher et al., 2018; Kelleher et al., 2012; Laurens et al., 2007 **)**. Consistent with these population‐based investigations, the current findings revealed strong associations between PQ‐B scores and increased ratings for both psychotic and nonpsychotic symptoms. The latter relationship conforms to prior research that has emphasized the importance of including nonpsychotic affective symptoms of depression and anxiety in risk formulation as well as measures of childhood adversity in nonhelp‐seeking samples (e.g., Linscott & Van Os, 2013; Yamasaki et al., 2018).

While providing strong support for extending the clinical dimensions of ARMs, the present investigation also provided clear evidence linking reduced psychiatric vulnerability to a set of measures aimed to tap distinct aspects of positive mental health ranging from plasticity genes, personality traits, and social cognition. In particular, behavioral data showed a consistent pattern of reduced ARMS, as measured by the PQ‐B, correlating strongly with better mental health, as assessed by MHC‐SF, and a distinct NEO personality profile of diminished neuroticism and elevated extraversion, agreeableness, and conscientiousness. In addition, reduced risk correlated with better performance on the RMET a widely used, sensitive measure of a key aspect of social cognition, known as the theory of mind. The theory of mind reflects a specific set of abilities that are distinct from cognitive intelligence and essential for the development of effective social interaction and communication (e.g., Heyes & Firth, 2014), and frequently lowered in at‐risk individuals (Fusar‐Poli et al., 2012). As such, these data suggested that well‐developed social cognitive abilities may reduce risk and enhance mental health.

These behavioral data also conformed well to the differential‐susceptibility hypothesis that specific serotonin and BDNF genes confer plasticity, thereby allowing carriers of these alleles greater sensitivity to environmental factors, whether negative or positive, as well as to a wider range of developmental outcomes. Of particular relevance is the idea that more plasticity alleles, the greater the likelihood of benefit from positive experiences and events. Indeed, neural plasticity is defined as a fundamental property of the brain that allows it to change in response to external input, learning, and training (Forsyth & Lewis, 2017). Here, we focused on the serotonin short and BDNF met allelic pair as plasticity genes with our results showing carriers of these polymorphisms with lowest ARMS and highest mental health. This pattern extended to individual differences in personality traits as assessed by the NEO, with serotonin short and BFNF‐met genotype again showing highest level of emotional stability as assessed by neuroticism.

Taken together, these polygenic analyses pointed to a clear pattern of reduced risk, adaptive personality traits, and positive mental health for carriers of serotonin short and BDNF met alleles. The data further showed this positive mental health effect may be moderated by epistatic, gene‐by‐gene interaction of serotonin and BDNF alleles. In line with the differential‐susceptibility hypothesis, such genetic variation may mediate the development of emotional processes that reduce risk and contribute to adaptive mental health. However, this hypothesis also emphasizes the importance of positive environmental influences in triggering plasticity genes. In this regard, the current study did not include an index of positive childhood experiences. And while carriers of the serotonin short and BDNF met allelic pair had lowest exposure to childhood adversity, whether they also had increased exposure to positive environmental experiences and events is unknown, and would need to be independently assessed in future studies of large representative samples.

Clinically, the current findings align well with recent treatment studies focusing on the enhancement of healthy personality either via psychopharmacology (e.g., Ilieva, 2015; Knutson et al., 1998) or psychological therapy (Armstrong & Rimes, 2016). In particular, these studies have targeted neuroticism as key for not only lowering psychiatric vulnerability but also in protecting public health, given neuroticism's well‐established role in a variety of mental and medical disorders as well as its large impact on overall quality of life (Barlow et al., 2014; Widiger et al., 2019). Consistent with these studies, here we showed reduced neuroticism corresponded with highest mental health and genetic plasticity. Finally, our findings also resonate with research calling for incorporating positive psychology interventions in the treatment of clients who are suffering with depression **(**Sin & Lyubormirsky, 2009 **)**. Our data, in fact, suggested that psychiatrically vulnerable individuals may be suitable candidates for interventions that aim not only to reduce risk and symptoms, but also to promote, specifically, mental health and subjective well‐being. Indeed, the results, while clearly supporting extending the clinical reach of the ARMS diagnosis, may provide an even greater contribution toward developing a positive psychology treatment protocol for enhancing illness resistance and well‐being for both psychiatric vulnerable and healthy individuals.

Finally, there are several important limitations of the current study. First is the relatively small sample and the lack of follow‐up data on the 100 participants. Indeed, the current findings linking ARMS with a host of behavioral and genetic protective factor would be greatly extended by longitudinal studies with larger samples that would provide greater statistical power to test the reliability and robustness of these contributions to psychiatric vulnerability and resilience. Similarly, the current study focused on the role of anxiety and depression in the ARMS, as assessed by the PQ‐B. In support of our hypothesis, multivariate analyses offered clear empirical support for extending PQ‐B defined ARMS to include both anxiety and depression. However, univariate correlational results, while linking anxiety and depression to PQ‐B, pointed to other psychiatric symptoms as also key contributors to risk and resilience.

## 5 CONCLUSIONS

In summary, the results provided support for not only extending the ARMS to include other psychiatric symptoms namely anxiety and depression, but also pointed to a number of protective factors that may reduce vulnerability and enhance mental health. Specifically, a personality configuration characterized by low neuroticism and elevated extraversion, agreeableness, and conscientiousness along with higher social cognitive abilities and greater positive mental health, as well as a particular set of BDNF and 5HTTLPR plasticity alleles may increase both illness resistance and well‐being.

## AUTHOR CONTRIBUTION

PGN, VCH, KOD, and RH contributed to the conception and design of this study. KOD, VCH, SBB, and HEL collected and organized the data. All authors analyzed the data, contributed to the drafting and editing of the manuscript and read and approved the final manuscript.

### PEER REVIEW

The peer review history for this article is available at https://publons.com/publon/10.1002/brb3.2137.

## Data Availability

The data that support the findings of this study are available from the corresponding author upon reasonable request.
